# Clinical improvement following therapy for periodontitis: Association with a decrease in IL-1 and IL-6

**DOI:** 10.3892/etm.2014.1724

**Published:** 2014-05-19

**Authors:** CÁTIA REIS, ALEXANDRA VIANA DA COSTA, JOÃO TIAGO GUIMARÃES, DIANA TUNA, ANA CRISTINA BRAGA, JOSÉ JULIO PACHECO, FERNANDO A. AROSA, FILOMENA SALAZAR, ELSA MARIA CARDOSO

**Affiliations:** 1 CESPU, Instituto de Investigação e Formação Avançada em Ciências e Tecnologias da Saúde, Gandra PRD 4585-116, Portugal; 2 Department of Clinical Pathology, Hospital de São João, Biochemistry Department, Faculty of Medicine, University of Porto, Al. Prof. Hernâni Monteiro, Porto 4200-319, Portugal; 3 Algoritmi Centre, Department of Production and Systems Engineering, School of Engineering, University of Minho, Campus de Gualtar, Braga 4710-057, Portugal; 4 Health Sciences Research Centre, Faculty of Health Sciences, University of Beira Interior, Covilhã 6200-506, Portugal

**Keywords:** periodontal therapy, biomarkers, cytokines, gingival crevicular fluid, multiplex immunoassay

## Abstract

Although a number of inflammatory cytokines have been shown to be associated with periodontal pathogenesis, it is important to investigate further whether these biomarkers are associated with the degree of success in nonsurgical treatment of chronic periodontitis. The aim of the present study was to quantify the total levels of interleukin (IL)-1α, -1β, -6, -10 and tumour necrosis factor (TNF)-α in gingival crevicular fluid (GCF) of chronic periodontitis patients prior to and following nonsurgical periodontal therapy. In total, 52 GCF samples from disease sites of patients with chronic periodontitis, prior to and following periodontal therapy, and ten non-disease sites from non-periodontitis subjects, were collected and cytokine concentrations were determined using a multiplex method. Periodontal parameters, including bleeding on probing, probing pocket depth and the clinical attachment level, in all the sites were recorded. Untreated disease sites exhibited higher cytokine levels in the GCF when compared with the non-disease sites. Nonsurgical periodontal therapy resulted in a statistically significant decrease in the total levels of IL-1α, -1β and -6 in the GCF, but not in IL-10 or TNF-α. The results support the hypothesis that proinflammatory cytokines, including IL-1α, IL-1β and IL-6, are likely to be involved in the pathogenesis of periodontitis and are good markers to evaluate the success of nonsurgical therapy in disease sites of patients with periodontitis.

## Introduction

Chronic periodontitis is a bacterial-induced chronic inflammation within the structures that support the teeth, resulting in progressive attachment and bone loss (1). Chronic periodontitis is considered to be a multifactorial disease, where clinical expression is determined by several environmental and host-derived risk factors, including microbial biofilm composition, and genetic background susceptibility or systemic disorders. Host behaviour, such as oral hygiene habits or smoking, also influence the course of the disease (2).

The chronic inflammatory response that occurs within the periodontal tissue is a complex process that involves innate and adaptive immune cells and their secreted molecules. It is currently accepted that proinflammatory cytokines produced locally by periodontal tissue and inflammatory immune cells contribute to disease progression (3), indicating them as putative periodontal disease biomarkers. Identification of molecular biomarkers that anticipate the degree of success of nonsurgical treatment may be of great benefit in clinical practice. In addition, such potential factors may aid the identification of tooth sites that have not improved at re-evaluation. According to the study by Kinane *et al*, periodontal disease biomarkers can be grouped into several categories, namely, prognostic biomarkers that identify patients or sites most likely to respond to a specific treatment, and therapeutic biomarkers that provide a quantifiable measurement of the response to periodontal treatment (4). To the best of our knowledge, no biomarker has been shown to exhibit a prognostic value at the disease site or at the patient level. With regard to therapeutic biomarkers, several studies have hypothesised that inflammatory cytokines, including interleukin (IL)-1 and tumour necrosis factor (TNF)-α, may be used as biomarkers to assess therapeutic outcomes in chronic periodontitis, based on studies where a reduction in inflammatory cytokines in the gingival crevicular fluid (GCF) was observed in response to nonsurgical periodontal therapy (3,4). However, these conclusions have not been corroborated in other studies (5–7). Numerous studies evaluating the level of proinflammatory cytokines in the GCF have included a diverse array of patients, including patients with moderate to advanced periodontitis, patients with aggressive periodontitis and patients with associated chronic and/or systemic diseases undergoing immunosuppressive therapy, which is known to influence immune parameters. The contrasting data highlights the need for further investigation. Thus, the aim of the present study was to investigate the influence of nonsurgical periodontal therapy on the levels of four typical proinflammatory cytokines, including IL-1α, -1β, -6 and TNF-α, as well as one anti-inflammatory cytokine, IL-10, in the GCF of patients with chronic periodontitis and no associated chronic pathologies. Correlation analysis was then performed with the clinical parameters of the disease.

## Materials and methods

### Study population

In total, 62 sampling sites were collected from subjects attending the Dental Sciences Clinic at the Department of Instituto Superior de Ciências da Saúde-Norte (Gandra, Portugal; ISCS-N). Informed consent was obtained from each patient prior to enrolment in the study and the experimental protocols were approved by the Ethics Committee of ISCS-N, according to the Declaration of Helsinki. The mean age of the subjects was 45.3±12.8 years, and all the subjects were Caucasian. In total, 81% were female and 19% were male. The subjects were non-smokers, with the exception of two periodontal disease subjects who smoked ≤10 cigarettes/day. Exclusion criteria included pregnancy or lactation, systemic diseases or intake of medication, such as antibiotics, anti-inflammatory agents or immunosuppressors, for six months prior to the study due to their possible effects on the immune or inflammatory response.

### Periodontal examination

All patients received a comprehensive periodontal examination, which included the determination of the probing pocket depth (PPD), bleeding on probing (BOP) and clinical attachment level (CAL). PPD determination was performed by measuring the gingival pocket (mm) using a graduated periodontal probe (CP11; ASA Dental, Bozzano Massarosa, Italy) at each surface of the teeth in the dentition (six sites per tooth: mesiobuccal, buccal, distobuccal, mesiolingual, lingual and distolingual). Measurements were performed starting from the free edge of the gum to the deep groove with the probe parallel to the long axis of the tooth. BOP during the measuring of previous parameters was present or absent, and BOP positive was considered an objective sign of gingival inflammation. CAL, which represented the clinical approach of the adhesion level of the tissue to the root surface, was evaluated using the same graduated probe, corresponding to the distance (mm) between the cemento-enamel junction and the deep groove.

### Periodontal treatment and re-evaluation

Following periodontal examination, patients with chronic periodontitis were enrolled in a nonsurgical periodontal treatment plan. Thus, the treatment provided to each patient consisted of scaling and root planning in the affected sites. Scaling comprised the removal of tartar infragingival and root planning on the surfaces of the teeth that had a PPD of ≥4 mm. Following the completion of treatment, follow-up (re-evaluation) was performed. The follow-up was performed once, 2 months after treatment

### Site selection and sample collection

In total, 52 samples were collected from disease sites (PPD, ≥4 mm) of chronic periodontal disease subjects and ten samples were collected from non-disease sites of subjects without periodontitis. Subjects received instruction to not eat, drink or brush the teeth for 1 h prior to GCF sampling. Prior to GCF sampling, the individual tooth was isolated with cotton rolls, supragingival plaque was carefully removed and the site was gently air-dried with an air syringe. A sterile paper point (Dentsply Maillefer, Tulsa, OK, USA) was inserted in each selected pocket until mild resistance was felt, left in the crevices for 30 sec and then immediately transferred into sterile eppendorf tubes, which were stored at −20°C until required for further analysis. In cases of visible contamination with blood, the paper point was discarded and a new site was selected. In periodontitis patients, GCF collection was performed at two points; the baseline prior to therapy and post-therapy at the periondontal re-evaluation.

### Processing GCF samples

For GCF cytokine determination, paper points were thawed, cut to 1 cm in length and thawed with 50 μl phosphate-buffered saline solution 1X [13 mM Na_2_HPO_4_, 7 mM NaHPO_4_, 100 mM NaCl (pH 7.0)] at 4°C overnight. Next, the paper points were centrifuged at 13,000 × g for 10 min at 4°C. Following centrifugation, 25-μl samples were used for cytokine evaluation with a multiplex immunoassay.

### Determination of cytokine levels

Cytokine (IL-1α, -1β, -6, -10 and TNF-α) concentrations were determined using a commercial multiplex fluorescent bead-based immunoassay kit (Human Cytokine/Chemokine Kit - MPXHCYTO-60K; Millipore Corporation, Billerica, MA, USA) in a Luminex^®^ 200™ analyser (Luminex Corporation, Austin, TX, USA). Raw data (mean fluorescence intensity) were analysed using IS™ 2.3 software (Luminex Corporation). Measurements were performed according to the manufacturer’s instructions, and standards and samples were measured in duplicate. The minimum detectable concentrations for each cytokine were 0.1 pg/ml for IL-1α, IL-10 and TNF-α, and 0.4 pg/ml for IL-1β and IL-6. Samples with concentrations below the limit of detection were scored as 0. Briefly, for the assay, 25-μl GCF samples were added to 25 μl assay buffer and incubated with anti-human multi-cytokine beads at 4°C for 18 h. Unbound material was removed by filtration. For revelation, 25 μl streptavidin-phycoerythrin was added, and incubated for 30 min. The reaction was stopped with 25 μl stop solution and plate reading occurred 15 min later. In the GCF samples, the total cytokine levels per site (pg/site) were determined with the assumption that all the cytokines present in the paper points were transferred to the phosphate-buffered saline solution.

### Statistical analysis

Statistical analysis was conducted using SPSS 20.0 (IBM, Armonk, NY, USA) software and P<0.05 was considered to indicate a statistically significant difference. Continuous variables with a normal distribution are expressed as the mean ± standard error of the mean and were analysed using parametric tests (T-test for paired or independent samples). Since the cytokine levels were not normally distributed, these data are expressed as the median (minimum and maximum) and were analysed using non-parametric tests (Mann-Whitney U test for unrelated samples or Wilcoxon signed-rank test for related samples). McNemar’s or Fisher’s exact tests were used to compare frequencies between related or unrelated samples, respectively. Spearman’s ρ correlation coefficient was used to analyse the correlations between clinical parameters and cytokine levels.

## Results

### Clinical parameters in the sample disease sites

Examination of the clinical parameters in the samples of untreated disease sites in the periodontitis patients revealed a worse clinical state when compared with the non-disease samples of patients without periodontitis (Table I). Thus, patients prior to treatment exhibited statistically significant increased PPD, CAL and BOP values (P<0.001, P<0.001 and P=0.005, respectively). Nonsurgical therapy resulted in a statistically significant decrease (P<0.001) in the PPD, from an average of 4.9 to 3.4 mm, as well as a decrease in the CAL from 1.9 to 0.7 mm (Table I). Following treatment, the sample sites from periodontitis patients revealed statistically significant higher PPD and CAL values when compared with the sample sites from non-disease sites. Although the percentage of BOP sites decreased between 59.6%, prior to treatment, to 40.4% following treatment, this decrease did not reach statistical significance (P=0.064).

### Cytokine levels in the GCF

Among the five cytokines analysed, IL-1α was the most prevalent cytokine found in the GCF and was detected in all the sites studied (Table II). By contrast, the majority of the other cytokine determinations were very low and in certain cases even below the detection levels, despite using a very sensitive method. Thus, considering all the GCF samples (from periodontitis patients and controls), the percentage of samples considered below the detection level were 13% for IL-1β, 31% for IL-6, 3% for IL-10 and 5% for TNF-α (data not shown). However, for all the cytokines studied, statistically significant higher levels were observed in the untreated disease sites when compared with the control non-disease sites (Table II). With the exception of IL-6, the difference between the patient and control sites was maintained following periodontal treatment.

Notably, following nonsurgical therapy, the total levels of the proinflammatory cytokines, IL-1α, -1β and -6, but not the anti-inflammatory cytokine IL-10, were significantly reduced. TNF-α, despite being proinflammatory, did not exhibit a significant decrease (Table II).

### Cytokine levels and clinical parameters in the disease sample sites

In order to ascertain the possible clinical relevance of these observations, correlation analysis between the clinical parameters and total cytokine levels in the GCF sample sites was performed. As shown in Table III, positive correlations were observed between the levels of IL-1α, -1β, -10 and TNF-α (but not IL-6) with PPD and CAL (Table III). However, no association was observed between the cytokine levels and BOP (data not shown).

## Discussion

Upon bacterial infection, gingival cells, including fibroblasts and epithelial cells, and cells of the immune system, such as macrophages and immature dendritic cells, present in the gingival/periodontal tissue, secrete a diverse array of cytokines that function as strong local mediators of inflammation to counteract infection. Among these, IL-1α, -1β, -6 and TNF-α are major players in the periodontal inflammatory process. Nonsurgical scaling and root planning are widely used as the therapy of choice for the effective treatment of moderated and advanced chronic periodontitis. In the present study, the total levels of IL-1α, -1β and -6 in the GCF of disease sites in chronic periodontitis patients decreased in response to nonsurgical therapy. By contrast, the levels of IL-10 and TNF-α did not change significantly. These results indicate that IL-1α, -1β and -6 may be involved in inflammation of the periodontal tissue.

The results indicating a decrease in the levels of the proinflammatory cytokines, IL-1α, -1β and -6, but not IL-10 or TNF-α, in the GCF of patients with chronic periodontitis, confirm the observations of previous studies. A previous study evaluating an extensive panel of GCF mediators, prior to and following initial therapy, in subjects with generalised severe chronic periodontitis, demonstrated that the total levels of numerous cytokines and chemokines, including IL-1α, -1β and -6, decreased significantly in disease sites in response to therapy (8). Additional studies revealed that following therapy, the level of IL-1β in the GCF was reduced (9), while the total level of IL-10 remained unchanged (10). In addition, a previous study did not identify a statistically significant difference in the total amount of TNF-α prior to and following periodontal treatment in chronic periodontitis subjects (11). Despite the apparent general consensus of a decrease in proinflammatory cytokines following nonsurgical therapy, certain studies have not produced such observations. In a study of 12 patients with moderate to advanced periodontitis, no statistically significant reduction in the total levels of IL-1β and -10 following nonsurgical therapy was observed (12). Furthermore, previous studies have demonstrated that total IL-1β levels were not decreased following therapy unless a subgroup of smokers were removed from the analysis (13,14). Finally, an additional study was unable to detect differences in the GCF levels of IL-1β and -6 following scaling and root planning (15). Thus, the results of the present study corroborate the existence of a close association between nonsurgical therapy and a significant decrease in the total amount of inflammatory cytokines, including IL-1α, -1β and -6. Whether the observed discrepancies between the studies are methodological, statistical or associated with the sample size or exclusion criteria requires further investigation. In this regard, it is important to consider that smoking is a putative factor that affects the levels of proinflammatory cytokines (13,14,16).

Overall analysis of the sample sites revealed a positive correlation between the levels of the proinflammatory cytokines, IL-1α and -1β, and the clinical severity, namely the PPD and CAL. These results strongly support the hypothesis that these cytokines are likely to be involved in the pathogenesis of periodontitis, as previously reported (12).

In conclusion, the present study supports and extends the observations of previous studies by demonstrating that the inflammatory cytokines, IL-1α and -1β, present in the GCF, correlate with clinical parameters, reinforcing the hypothesis that these cytokines are important markers in the pathogenesis of chronic periodontitis. The study also indicates that evaluating the total levels of inflammatory cytokines in the GCF of periodontitis patients may be a useful laboratory test to monitor the response to nonsurgical treatment.

## Figures and Tables

**Table I:** Clinical sample site parameters.

Parameter	Disease sites (n=52)		P-value	Non-disease sites (n=10)	P-value	P-value

	Prior to treatment	Following treatment				
PPD (mm)	4.9±0.1	3.4±0.2	<0.001^a^	0.5±0.2	<0.001^c^	<0.001^d^
CAL (mm)	1.9±0.2	0.7±0.1	<0.001^a^	0.0±0.0	<0.001^c^	<0.001^d^
BOP sites (n)	31	21		1		
BOP sites (%)	59.6	40.4	0.064^b^	10.0	0.005^e^	0.082^f^

Statistics were calculated using

a the Paired Samples T-Test,

b McNemar Test,

c Independent Samples T-Test

c comparison between disease sites prior to treatment and non-disease sites;

d comparison between disease sites following treatment and non-disease sites, and Fisher’s Exact Test

e comparison between disease sites prior to treatment and non-disease sites;

f comparison between disease sites following treatment and non-disease sites.

P<0.05 indicates a statistically significant difference. PPD and CAL values are expressed as the mean ± SEM. BOP, bleeding on probing; CAL, clinical attachment level; PPD, probing pocket depth.

**Table II:** Cytokine levels in the GCF samples.

Cytokine (pg/site)	Disease sites (n=52)		P-value^a^	Non-disease sites (n=10)	P-value^b^	P-value^c^

	Prior to treatment	Following treatment				
IL-1α	72.03 (2.17–2099.89)	29.70 (0.75–541.85)	0.001	11.55 (8.05–46.25)	<0.001	0.007
IL-1β	0.57 (0.00–126.95)	0.09 (0.00–35.15)	0.007	0.01 (0.00–0.14)	<0.001	0.014
IL-6	0.13 (0.00–2.32)	0.06 (0.00–1.16)	0.047	0.00 (0.00–0.28)	0.004	0.056
IL-10	0.13 (0.00–1.46)	0.07 (0.00–0.68)	0.257	0.01 (0.00–0.05)	<0.001	<0.001
TNF-α	0.06 (0.01–0.52)	0.04 (0.00–0.45)	0.243	0.01 (0.00–0.13)	0.005	0.049

Statistics were calculated using

a related-Samples Wilcoxon Signed Rank Test and independent-Samples Mann Whitney U Test

b comparision between disease sites prior to treatment and non-disease sites;

c comparison between disease sites following treatment and non-disease sites.

P<0.05 indicated a statistically significant difference. Results are expressed as the median (minimum-maximum). GCF, gingival crevicular fluid; IL, interleukin; TNF, tumour necrosis factor.

**Table III:** Correlation analysis between clinical parameters (mm) and cytokine levels (pg/site) in GCF sample sites (n=62).

PPD correlation	ρ^a^	P-value	CAL correlation	ρ^a^	P-value
IL-1α	0.386	0.002	IL-1α	0.390	0.002
IL-1β	0.437	<0.001	IL-1β	0.439	<0.001
IL-6	0.230	0.072	IL-6	0.238	0.062
IL-10	0.457	<0.001	IL-10	0.460	<0.001
TNF-α	0.262	0.039	TNF-α	0.275	0.030

a Spearman’s ρ correlation coefficient; P<0.05 indicated a statistically significant difference.

CAL, clinical attachment level; GCF, gingival crevicular fluid; PPD, probing pocket depth; IL, interleukin; TNF, tumour necrosis factor.

## References

[1] Lindhe J, Ranney R, Lamster I (1999). Consensus report: Chronic periodontitis. Ann Periodontol.

[2] Stabholz A, Soskolne WA, Shapira L (2010). Genetic and environmental risk factors for chronic periodontitis and aggressive periodontitis. Periodontol 2000.

[3] Buduneli N, Kinane DF (2011). Host-derived diagnostic markers related to soft tissue destruction and bone degradation in periodontitis. J Clin Periodontol.

[4] Kinane DF, Preshaw PM, Loos BG, Working Group 2 of Seventh European Workshop on Periodontology (2011). Host-response: understanding the cellular and molecular mechanisms of host-microbial interactions - consensus of the Seventh European Workshop on Periodontology. J Clin Periodontol.

[5] Yoshinari N, Kawase H, Mitani A (2004). Effects of scaling and root planing on the amounts of interleukin-1 and interleukin-1 receptor antagonist and the mRNA expression of interleukin-1beta in gingival crevicular fluid and gingival tissues. J Periodontal Res.

[6] Oringer RJ, Al-Shammari KF, Aldredge WA (2002). Effect of locally delivered minocycline microspheres on markers of bone resorption. J Periodontol.

[7] Giannopoulou C, Cappuyns I, Cancela J, Cionca N, Mombelli A (2012). Effect of photodynamic therapy, diode laser, and deep scaling on cytokine and acute-phase protein levels in gingival crevicular fluid of residual periodontal pockets. J Periodontol.

[8] Thunell DH, Tymkiw KD, Johnson GK (2010). A multiplex immunoassay demonstrates reductions in gingival crevicular fluid cytokines following initial periodontal therapy. J Periodontal Res.

[9] Tüter G, Kurtiş B, Serdar M (2001). Interleukin-1beta and thiobarbituric acid reactive substance (TBARS) levels after phase I periodontal therapy in patients with chronic periodontitis. J Periodontol.

[10] Goutoudi P, Diza E, Arvanitidou M (2004). Effect of periodontal therapy on crevicular fluid interleukin-1beta and interleukin-10 levels in chronic periodontitis. J Dent.

[11] Ikezawa-Suzuki I, Shimada Y, Tai H, Komatsu Y, Tanaka A, Yoshie H (2008). Effects of treatment on soluble tumour necrosis factor receptor type 1 and 2 in chronic periodontitis. J Clin Periodontol.

[12] Gamonal J, Acevedo A, Bascones A, Jorge O, Silva A (2000). Levels of interleukin-1 beta, -8, and -10 and RANTES in gingival crevicular fluid and cell populations in adult periodontitis patients and the effect of periodontal treatment. J Periodontol.

[13] Rosalem W, Rescala B, Teles RP, Fischer RG, Gustafsson A, Figueredo CM (2011). Effect of non-surgical treatment on chronic and aggressive periodontitis: clinical, immunologic, and microbiologic findings. J Periodontol.

[14] Al-Shammari KF, Giannobile WV, Aldredge WA (2001). Effect of non-surgical periodontal therapy on C-telopeptide pyridinoline cross-links (ICTP) and interleukin-1 levels. J Periodontol.

[15] Kardeşler L, Buduneli N, Çetinkalp S, Lappin D, Kinane DF (2011). Gingival crevicular fluid IL-6, tPA, PAI-2, albumin levels following initial periodontal treatment in chronic periodontitis patients with or without type 2 diabetes. Inflamm Res.

[16] Tymkiw KD, Thunell DH, Johnson GK (2011). Influence of smoking on gingival crevicular fluid cytokines in severe chronic periodontitis. J Clin Periodontol.

